# A Glucuronic Acid-Palmitoylethanolamide Conjugate (GLUPEA) Is an Innovative Drug Delivery System and a Potential Bioregulator

**DOI:** 10.3390/cells10020450

**Published:** 2021-02-20

**Authors:** Emiliano Manzo, Aniello Schiano Moriello, Francesco Tinto, Roberta Verde, Marco Allarà, Luciano De Petrocellis, Ester Pagano, Angelo A. Izzo, Vincenzo Di Marzo, Stefania Petrosino

**Affiliations:** 1 Istituto di Chimica Biomolecolare, CNR, 80078 Pozzuoli, Napoli, Italy; emanzo@icb.cnr.it (E.M.); aniello.schianomoriello@icb.cnr.it (A.S.M.); francesco.tinto.1@ulaval.ca (F.T.); roberta.verde@icb.cnr.it (R.V.); mallara@icb.cnr.it (M.A.); luciano.depetrocellis@icb.cnr.it (L.D.P.); 2 Endocannabinoid Research Group, 80078 Pozzuoli, Napoli, Italy; ester.pagano@unina.it (E.P.); aaizzo@unina.it (A.A.I.); 3 Epitech Group S.p.A., 35030 Saccolongo, Padova, Italy; 4 Dipartimento di Farmacia, Università di Napoli Federico II, 80138 Naples, Napoli, Italy; 5 Canada Excellence Research Chair on the Microbiome-Endocannabinoidome Axis in Metabolic Health, CRIUCPQ and INAF-Centre NUTRISS, Faculties of Medicine and Agriculture and Food Sciences, Université Laval, Quebéc, QC G1V 0A6, Canada

**Keywords:** allergic contact dermatitis, chemokine, colitis, drug-carrier, endocannabinoid system, inflammation, keratinocytes, palmitoylethanolamide, pro-drug, TRPV1

## Abstract

Palmitoylethanolamide (PEA) is an endogenous anti-inflammatory lipid mediator and a widely used nutraceutical. In this study, we designed, realized, and tested a drug-carrier conjugate between PEA (the active drug) and glucuronic acid (the carrier). The conjugate, named GLUPEA, was characterized for its capability of increasing PEA levels and exerting anti-inflammatory activity both in vitro and in vivo. GLUPEA treatment, compared to the same concentration of PEA, resulted in higher cellular amounts of PEA and the endocannabinoid 2-arachidonoyl glycerol (2-AG), and increased 2-AG-induced transient receptor potential vanilloid type 1 (TRPV1) channel desensitization to capsaicin. GLUPEA inhibited pro-inflammatory monocyte chemoattractant protein 2 (MCP-2) release from stimulated keratinocytes, and it was almost as efficacious as ultra-micronized PEA at reducing colitis in dinitrobenzene sulfonic acid (DNBS)-injected mice when using the same dose. GLUPEA is a novel pro-drug able to efficiently mimic the anti-inflammatory and endocannabinoid enhancing actions of PEA.

## 1. Introduction

Palmitoylethanolamide (PEA) is a substance of natural origin produced not only in many plant and animal food sources [1,2,3,4,5,6,7], but also in a broad variety of mammalian cells and tissues [8,9,10,11,12,13,14,15,16,17,18,19]. Chemically, PEA is the amide of a fatty acid—palmitic acid—with ethanolamine. One of the main enzymes responsible for its biosynthesis is *N*-acyl-phosphatidyl-ethanolamine-selective phospholipase D (NAPE-PLD), which catalyzes the hydrolysis of direct PEA phospholipid precursors, i.e., *N*-palmitoyl-phosphatidyl-ethanolamines [20]. The hydrolysis of PEA to palmitic acid and ethanolamine depends on the catalytic action of both fatty acid amide hydrolase (FAAH) [21] and, more specifically, *N*-acylethanolamine-hydrolyzing acid amidase (NAAA) [22].

**Scheme 1:**
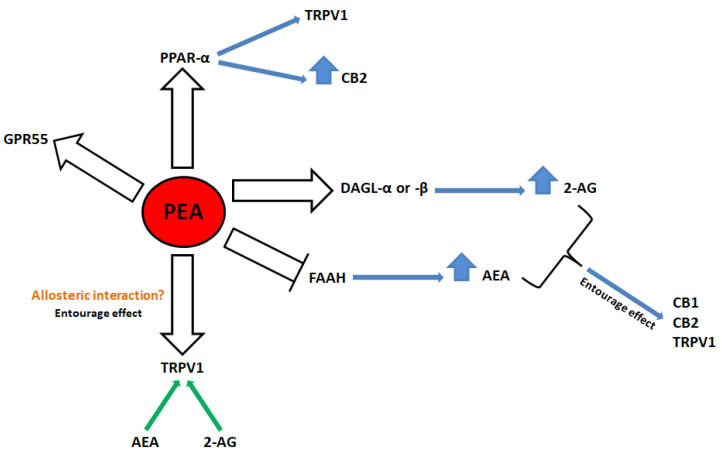
Palmitoylethanolamide (PEA) mechanism of action. PEA has been suggested to directly activate GPR55 [24], or peroxisome proliferator-activated receptor-α (PPAR-α) [23], and, through the activation of PPAR-α, to sensitize transient receptor potential vanilloid type 1 (TRPV1) channels or increase the expression of cannabinoid receptor type 2 (CB2) [25,26,27]. PEA, by stimulating the activity of diacylglycerol lipase (DAGL)-α and -β [29], or by inhibiting the expression of fatty acid amide hydrolase (FAAH) [28], indirectly activates cannabinoid receptor type 1 (CB1) and CB2, or TRPV1 channels. PEA, possibly through an allosteric interaction, may also enhance anandamide (AEA)- or 2-arachidonoyl glycerol (2-AG)-induced TRPV1 channel activation and desensitization [28,35,36,37].

The mechanism of action of PEA is still under investigation. It seems that PEA does not act through just one main mechanism, but conversely, via synergistic interactions among different signaling pathways, thus explaining its pleiotropic therapeutic effects [7,19]. In fact, both direct and indirect (via “entourage” effects) receptor-mediated mechanisms have been demonstrated for this mediator. In particular, it has been proved that PEA is able to directly activate the peroxisome proliferator-activated receptor-α (PPAR-α) [23], or the orphan receptor G-protein coupled GPR55 [24], and, through the activation of PPAR-α, activate transient receptor potential vanilloid type 1 (TRPV1) channels or increase the expression of the cannabinoid receptor type 2 (CB2) [25,26,27]. Additionally, PEA, by inhibiting the expression of FAAH [28] or by stimulating the activity of diacylglycerol lipase (DAGL-α and -β, the biosynthetic enzymes for 2-arachidonoyl-glycerol (2-AG)) [29], may indirectly activate cannabinoid receptors of type-1 (CB1) and type-2 (CB2), or TRPV1 channels, which are alternative targets for the endocannabinoids, anandamide (AEA) and 2-AG (Scheme 1) [30,31,32,33,34]. Finally, PEA, probably through an allosteric interaction, is also capable of enhancing AEA- or 2-AG-induced TRPV1 channel activation and desensitization [28,35,36,37]. 

Although there is an extensive body of literature on the anti-neuro-inflammatory and analgesic actions of PEA [18,19,37], there are only a few papers reporting data on the pharmacokinetics of this compound [7,38]. Early findings suggest a low bioavailability for this compound, due to its low solubility in water, which limits its absorption following oral administration [39,40]. Accordingly, Artamonov and colleagues (2005) demonstrated that even though *N*-[9,10-^3^H]-PEA accumulates in the hypothalamus, pituitary, and adrenal glands of rats 20 min following oral administration, its levels were very low [39]. Likewise, Vacondio and colleagues (2005) showed that when PEA (at a dose of 100 mg kg^−1^) was suspended in corn oil and orally administered to rats, the highest concentration was achieved in the plasma after 15 min and then decreased 2 h following administration [40]. Accordingly, in order to improve the oral bioavailability of PEA, particle size reduction has been considered because it is well-known that such an approach increases the specific surface area, thereby enhancing the solubility and improving the bioavailability [41]. Therefore, two new formulations of PEA were realized, i.e., micronized and ultra-micronized PEA (PEA-m and PEA-um) [7,19,38]. Both formulations were able to increase plasma PEA levels up to 2-fold 2 h after the administration of PEA-m (300 mg/tablet, ~5 mg kg^−1^) in human volunteers, or up to 6-fold 2 h after the administration of PEA-um (30 mg kg^−1^) in beagle dogs [37]. Importantly, the increased plasma PEA levels were accompanied by a significant increase of the plasma levels of the endocannabinoid 2-AG, which increased up to 2-fold 4 and 6 h after the administration of PEA-m, or up to 20-fold 2 h after the administration of PEA-um [37]. More recently, it has also been proven that rats orally administered [^13^C]_4_-PEA-um (30 mg kg^−1^) displayed low-medium nanomolar levels of [^13^C]_4_-PEA in the brain shortly following administration [42]. In addition, in a subsequent study, the overall plasma concentrations were higher in either healthy or carrageenan-injected rats administered [^13^C]_4_-PEA-um compared to those receiving naïve [^13^C]_4_-PEA, demonstrating the greater absorbability of PEA-um [38]. Additionally, a carrageenan injection strongly favored an increase in concentrations of [^13^C]_4_-PEA in the spinal cord and paw [38]. These data, altogether, suggest that PEA-m, and particularly PEA-um, represent a valuable strategy for overcoming PEA solubility problems.

Another strategy for improving the bioavailability of hydrophobic drugs is to develop drug-carrier conjugates, i.e., drugs chemically modified with carriers. In fact, it has been demonstrated that their conjugation to carriers may improve some properties of the drugs, including the lipophilicity, oral bioavailability, targeting of the lymphatic system, tumor targeting, efficacy, and safety [43,44,45]. In particular, the use of hydrophilic polymers for drug conjugation, such as poly(ethylene glycol), poly(zwitterionic), polypeptides, and polysaccharides, has received great attention as a method for the improvement of drug pharmacokinetics [46,47,48,49]. In this regard, hyaluronic acid, which is a naturally occurring polysaccharide, has emerged as an excellent carrier due to its high hydrophilicity and biocompatibility, and lack of toxicity [50,51,52].

Considering this background, the aim of the present study was to use glucuronic acid, which is one of the main hyaluronic acid constituents along with *N*-glucosamine, as the starting point to develop a new drug-carrier conjugate characterized by a homogeneous chemical composition. Therefore, we designed, realized, and tested a drug-carrier conjugate, named GLUPEA, between PEA (the active drug) and glucuronic acid (the carrier). GLUPEA was first characterized in in vitro cell systems to evaluate the release of the active drug—PEA—and then tested in an experimental model of allergic contact dermatitis (ACD) in human keratinocytes, and against colitis in mice, to evaluate its potential anti-inflammatory activity. Importantly, the mechanism of action and molecular targets of GLUPEA were also investigated in in vitro cell systems.

## 2. Materials and Methods

### 2.1. Reagents

All reagents were purchased from Sigma-Aldrich (Milano, Italy) unless otherwise specified. TLC plates (Kieselgel 60 F254) and silica gel powder (Kieselgel 60, 0.063–0.200 mm) were purchased from Merck (Darmstadt, Germany). All reagents for synthesis were used without any further purification. Synthetic intermediates and GLUPEA were characterized by NMR analysis. 1D- and 2D-NMR spectra were recorded on a Bruker Avance-400 (400.13 MHz) and on a Bruker DRX-600 equipped with a TXI CryoProbe in CDCl_3_ (δ values refer to CHCl_3_ at 7.26 ppm). HRESIMS was conducted on a Micromass Q-TOF micro. The human keratinocyte (HaCaT) cell line was purchased from CLS Cell Lines Service (Eppelheim, Germany). The human embryonic kidney (HEK-293) cell line was purchased from LGC Standards (Milano, Italy). PEA-um was provided by the Epitech Group S.p.A. (Saccolongo, Padova, Italy). Deuterated standards—[^2^H]_4_-PEA and [^2^H]_5_-2-AG—were purchased from Cayman Chemical (Cabru, Arcore, Italy). Capsaicin, ionomycin, and unlabeled 2-AG were purchased from ENZO Life Sciences (Roma, Italy). Methyl ester Fluo-4 (Fluo-4-AM) and Pluronic F-127 were purchased from Thermo-Fischer Scientific (Life Technologies Italia, Monza, Italy). Polyinosinic-polycytidylic acid [Poly-(I:C)] was purchased from InvivoGen (Aurogene, Roma, Italy). The monocyte chemoattractant protein 2 (MCP-2) Human ELISA Kit was purchased from RayBiotech, Inc. (Tebu-Bio, Magenta, Milano, Italy). Male ICR mice (25–30 g) were purchased from Harlan Italy (Correzzana, Monza Brianza, Italy). 

### 2.2. Procedure for the Synthesis of the Conjugate between PEA and Glucuronic Acid—GLUPEA

#### 2.2.1. Intermediate 1 

**Scheme 2:**
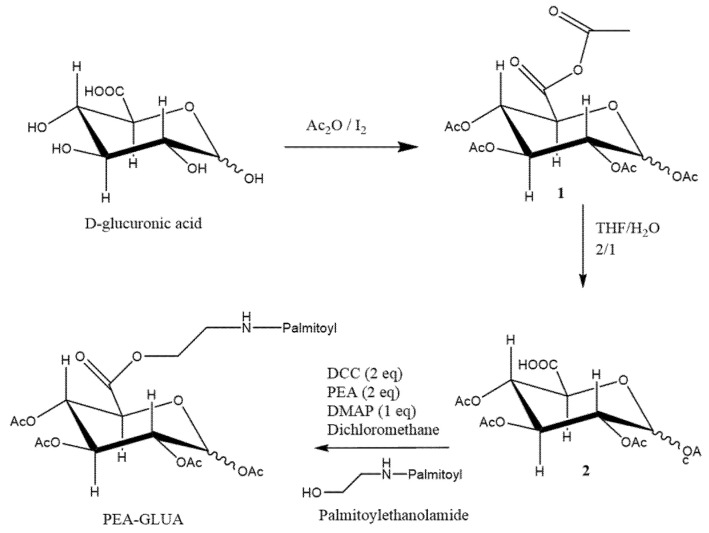
Chemical synthesis of the drug-carrier conjugate GLUPEA.

D-glucuronic acid (2.5 g, 12.6 mmoL) was dissolved in acetic anhydride (37 mL) and stirred at 0 °C; the iodine (175 mg, 0.83 mmoL) was slowly added and the mixture was stirred for 2 h at room temperature. After the evaporation of acetic anhydride in vacuo, the residue was dissolved in dichloromethane (35 mL). The organic phase was washed twice with Na_2_S_2_O_3_ (1 M, 40 mL), dried with MgSO_4_, filtered, and concentrated to give intermediate **1** (5.05 g, 98%) as an α and β anomer mixture (1/4) (Scheme 2); ^1^H-NMR (400 MHz, CDCl_3_): δ 6.29 (1 H, bs, α-form), 5.72 (1 H, d, J = 6.8 Hz, β-form), 5.45 (1 H, t, J = 9.5 Hz, α-form), 5.29–5.15 (2 H, overlapped, α- and β-form), 5.09 (1 H, t, J = 7.5 Hz, β-form), 4.38 (1 H, d, J = 9.2 Hz, α-form), 4.21 (1 H, d, J = 9.3 Hz β-form), 2.18–1.92 (15 H, -COCH_3_); HRESIMS m/z 427.0843 [M + Na]^+^ (calcd for C_16_H_20_O_12_Na, 427.0852). 

#### 2.2.2. Intermediate 2

Intermediate 1 (4.8 g, 12.5 mmoL) was dissolved in water and tetrahydrofuran (1/2, 90 mL) and stirred overnight at room temperature; the reaction mixture was concentrated and extracted in dichloromethane, dried with MgSO_4_, and filtered. After evaporation, intermediate 2 was obtained (3.9 g, 90%) (Scheme 2); ^1^H-NMR (400 MHz, CDCl_3_): δ 5.79 (1 H, d, J = 7.5 Hz), 5.30 (2 H, overlapped), 5.13 (1 H, m), 4.23 (1 H, d, J = 9.4 Hz), 2.12–1.94 (12 H, -COCH_3_); HRESIMS m/z 385.0755 [M + Na]^+^ (calcd for C_14_H_18_O_11_Na, 385.0747). 

#### 2.2.3. GLUPEA

Intermediate 2 (3.0 g, 8.2 mmol) was dissolved in dichloromethane (65 mL); *N*,*N*-dicyclohexylcarbodiimide (DCC) (3.3 g, 16.4 mmoL), *N*,*N*-dimethylaminopyridine (DMAP) (1.0 g, 8.2 mmoL), and palmitoylethanolamide (PEA) (16.4 mmol) were added. The mixture was then stirred overnight at room temperature. After the evaporation of solvent, the residue was portioned between water and diethylether and the organic phase was purified on a silica-gel column (eluent: petroleum ether/chloroform gradient), to finally obtain GLUPEA (1.9 g, 2.93 mmol, 36%) (Scheme 2); ^1^H-NMR (400 MHz, CDCl_3_): δ 6.39 (1 H, d, J = 3.6 Hz), 6.05 (1 H, bs), 5.77 (1 H, d, J = 7.4 Hz), 5.53 (1 H, t, J = 9.6 Hz), 5.35–5.26 (2 H, overlapped), 5.18–5.14 (2 H, overlapped), 4.44 (1 H, d, J = 9.03 Hz), 4.32 (1 H, m), 4.23–4.15 (2 H, overlapped), 3.62–3.48 (2 H, overlapped), 2.21 (2 H, t, J = 6.8 Hz), 2.20–2.05 (12 H, -COCH3); HRESIMS m/z 666.3472 [M+Na]^+^ (calcd for C_32_H_53_NO_12_Na, 666.3465). 

### 2.3. Cell Cultures

HaCaT cells were grown in Dulbecco’s Modified Eagle Medium (DMEM) complemented with glutamine (2 mM), penicillin (400 U mL^−1^), streptomycin (50 mg mL^−1^), and 10% Fetal Bovine Serum (FBS), in the presence of 5% CO_2_ atmosphere at 37 °C, plated on 100 mm diameter Petri dishes. 

HEK-293 cells, both wild type and stably transfected with human recombinant TRPV1 cDNA (HEK-TRPV1), were grown in Eagle’s Minimum Essential Medium (EMEM) complemented with glutamine (2 mM), non-essential amino acids, and 10% FBS, in the presence of 5% CO_2_ at 37 °C, plated on 100 mm diameter Petri dishes. HEK-293 cells stably transfected were selected using geneticin (G-418, 600 μg mL^−1^).

### 2.4. Analysis of PEA and 2-AG Levels by LC-APCI-MS

HaCaT cells were plated in 6-well culture dishes at a cell density of 9 × 10^5^ cells per well, and after 24 h, were treated with GLUPEA (10–20 μM), PEA (10–20 μM), vehicle (methanol 0.05%), or glucuronic acid (10–20 μM) and incubated for 40 min, 6 h, and 24 h at 37 °C in the presence of 5% CO_2_. After the stimulation, cell pellets or cells and supernatants were collected and homogenized in a solution of chloroform/methanol/Tris-HCl 50 mM pH 7.4 (2:1:1 by vol.) containing 5 pmol of [^2^H]_4_-PEA and [^2^H]_5_-2-AG as internal standards [32,53]. The lipid-containing organic phase was dried, weighed, and pre-purified by open-bed chromatography on silica gel. Fractions derived by eluting the column with a solution of chloroform/methanol (90:10 by vol.) were analyzed by Liquid Chromatography−Atmospheric Pressure Chemical Ionization−Mass Spectrometry (LC-APCI-MS) by using a Shimadzu (Shimadzu, Kyoto, Japan) HPLC apparatus (LC-10ADVP) coupled to a Shimadzu (LCMS-2020) quadrupole MS via a Shimadzu APCI interface. LC-APCI-MS analyses of PEA and 2-AG were performed in the selected ion monitoring mode [28,54], using m/z values of 304 and 300 (molecular ions +1 for deuterated and undeuterated PEA) and 384.35 and 379.35 (molecular ions +1 for deuterated and undeuterated 2-AG), respectively. PEA and 2-AG concentrations were determined on the basis of their area ratio with the internal deuterated standard signal areas, and their amounts (pmol) were normalized per mg of lipid extract.

### 2.5. Detection of Intracellular Calcium Elevation

HEK-TRPV1 cells were loaded for 1 h at room temperature with the selective intracellular fluorescent probe for Ca^2+^ ions (Fluo-4-AM, 4 μM), containing 0.02% Pluronic F-127 in EMEM without FBS. Instead, in HEK-TRPV1 cells pre-treated for 6 h with GLUPEA (20 μM), the selective intracellular fluorescent probe for Ca^2+^ ions (Fluo-4-AM, 4 μM) was loaded in the last hour of pre-treatment. 

After the incubation, cells were washed twice in Tyrode’s buffer (145 mM NaCl, 2.5 mM KCl, 1.5 mM CaCl_2_, 1.2 mM MgCl_2_, 10 mM D-glucose, and 10 mM HEPES, pH 7.4), resuspended in Tyrode’s buffer, and transferred to the quartz cuvette of the spectrofluorimeter (Perkin-Elmer LS50B; Perkin-Elmer Life, Waltham, MA, USA) under continuous stirring. Experiments were carried out by measuring cell fluorescence at 25 °C (excitation λ = 488 nm; emission λ = 516 nm) before and after the addition of compounds. The effects of GLUPEA or 2-AG on TRPV1 channels were calculated by normalizing their effects to the maximum Ca^2+^ influx effect on [Ca^2+^]_i_ obtained after the application of 4 μM ionomycin. Potency was expressed as the concentration of 2-AG or GLUPEA exerting the half-maximal agonistic effect (i.e., half-maximal increases in [Ca^2+^]_i_) (EC_50_). Desensitizing behavior was valued against the TRPV1 agonist capsaicin (0.1 μM) by adding 2-AG or GLUPEA at different concentrations in the quartz cuvette containing cells 5 min before the stimulation of cells with 0.1 μM capsaicin. Data are expressed as the concentration exerting a half-maximal inhibition of agonist-induced [Ca^2+^]_i_ elevation (IC_50_). The effect on [Ca^2+^]_i_ exerted by 0.1 μM capsaicin alone was taken as 100%. GLUPEA was also added to the cells 5 min before 2-AG to study its effect on 2-AG-induced activation of the TRPV1-mediated elevation of intracellular Ca^2+^ or on 2-AG desensitizing behavior. In order to investigate the effect of a longer incubation of cells with GLUPEA on TRPV1 activation induced by exogenous and endogenous TRPV1 agonists, and under conditions in which the effect on PEA levels could be evaluated, the compound was also added to HEK-TRPV1 cells 6 h before either 2-AG or capsaicin.

### 2.6. Poly-(I:C)-Induced ACD in HaCaT Cells

HaCaT cells were plated into 24-well culture dishes at a cell density of 2 × 10^5^ cells per well, and after 24 h, were stimulated with poly-(I:C) (100 μg mL^−1^) or vehicle (water) and incubated for 6 h at 37 °C in 5% CO_2_ [15]. The effect of GLUPEA was studied by treating poly-(I:C)-stimulated HaCaT cells with GLUPEA (20 μM), PEA (10 μM), or vehicle (methanol) for the indicated times. 

### 2.7. ELISA Assay

An enzyme-linked immunosorbent assay was performed after 6 h to quantify the MCP-2 chemokine concentration from cell supernatants obtained from poly-(I:C)-stimulated HaCaT keratinocytes in the presence of GLUPEA, PEA, or vehicle. The human MCP-2 ELISA kit protocol was used, according to the manufacturer’s instructions (RayBiotech). Data are expressed as the pg mL^−1^ of MCP-2.

### 2.8. Animals

All of the experimental procedures and protocols were in conformity with the ethics of laboratory animal care, in compliance with national (*Direttiva* 2010/63/UE) laws and policies and approved by the Italian Ministry of Health. The study on dinitrobenzene sulfonic acid (DNBS)-induced colitis in mice was approved by the Animal Care and Use Committee of the University of Naples Federico II. 

Male ICR mice, weighing 25–30 g, were used in the experiments described here. A total of 15 mice for each experimental group were used in this study. Animals were housed in polycarbonate cages under specific-pathogen-free conditions with a 12 h light/dark cycle and controlled temperature (23 ± 2 °C) and humidity (60%). Animals were fed ad libitum with standard food, except for the 24 h period immediately preceding the administration of DNBS and for the 2 h period preceding the administration of GLUPEA or PEA-um.

### 2.9. DNBS-Induced Colitis in Mice

Colitis was induced by an intracolonic injection of DNBS, as described previously [55]. Briefly, mice were anaesthetized with inhaled 5% isoflurane (Centro Agrovete Campania, Scafati, Salerno, Italy) and successively, DNBS (150 mg kg^−1^) was injected into the colon using a polyethylene catheter (1 mm in diameter) via the rectum (4.5 cm from the anus). Control mice received an intracolonic injection of water. Three days following DNBS or water injection, all animals were killed by asphyxiation with CO_2_. Then, the abdomen of mice was opened by a midline incision and the colon was removed, isolated from surrounding tissues, opened along the anti-mesenteric border, rinsed, and weighed and the length was measured, in order to determine the colon weight/colon length ratio (mg cm^−1^). The body weight of mice was measured every day throughout the treatment period. All measurements were performed by workers who were unaware of the particular treatment. The dose of DNBS was decided on the basis of preliminary experiments showing colonic damage associated with a high reproducibility and low mortality for a 150 mg kg^−1^ dose. The time point of damage evaluation (i.e., 3 days after DNBS was injected) was chosen because the maximal DNBS-induced inflammation has been reported in mice after 3 days [56]. GLUPEA (1–10 mg kg^−1^) or PEA-um (1–10 mg kg^−1^) was administered by oral gavage three times (once a day) for three consecutive days starting 24 h after DNBS or vehicle injection. Animals were killed 2 h after the third administration of GLUPEA or PEA-um. DNBS was dissolved in 50% ethanol. GLUPEA or PEA-um was suspended in carboxymethyl cellulose (1.5%, 150 μL per mouse) for oral administration. 

### 2.10. Myeloperoxidase (MPO) Activity

MPO activity was determined as previously described [55]. Full-thickness colons were homogenized in an appropriate lysis buffer (0.5% hexadecyltrimethylammonium bromide in 3-(*N* morpholino)propanesulfonic acid [MOPS] 10 mM) at the ratio of 50 mg tissue per mL MOPS. The samples were then centrifuged for 20 min at 15,000× *g* at 4 °C. Following this, an aliquot of the supernatant was incubated with NaPP (sodium phosphate buffer pH 5.5) and 16 mM tetra-methylbenzidine. After 5 min, H_2_O_2_ (9.8 M) in NaPP was added and the reaction was stopped with acetic acid. The rate of change in absorbance was measured by a spectrophotometer at 650 nm. Different dilutions of human MPO enzyme of a known concentration were used to obtain a standard curve. The MPO activity was expressed as U mg^−1^ of tissue.

### 2.11. Statistics

Data are expressed as means ± standard error of the mean (s.e.m.). For the experiments performed on HaCaT cells, the results were analyzed by one-way analysis of variance (ANOVA) followed by the Newman–Keuls multiple comparison test. For the experiments performed on the intracellular calcium elevation, concentration–response curves were fitted by a sigmoidal regression with a variable slope and statistics were calculated by ANOVA followed by Bonferroni’s multiple comparison test or by Student’s *t* test. For the experiments performed in mice, the data were analyzed by ANOVA followed by Dunnett’s multiple comparisons test. All data sets subjected to statistical analysis were compiled from four or more independent determinations. In all statistical analyses, a *p* value < 0.05 was considered as statistically significant. Statistics, curve fitting, and parameter evaluation were executed using GraphPad Prism version 8.0 (GraphPad Software Inc., San Diego, CA, USA). 

## 3. Results

### 3.1. GLUPEA Releases PEA in HaCaT Keratinocytes

**Figure 1:**
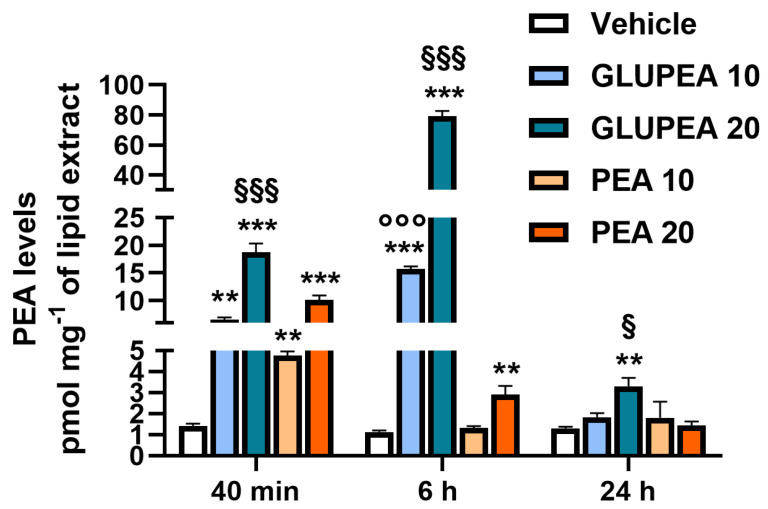
PEA levels in human keratinocyte (HaCaT) keratinocytes after glucuronic acid-palmitoylethanolamide conjugate (GLUPEA) treatment. PEA levels were measured in HaCaT keratinocytes treated for 40 min, 6 h, and 24 h with GLUPEA (10–20 μM) or PEA (10–20 μM). Data are representative of *n* = 8 separate determinations (means + s.e.m.). Statistical analyses were carried out using one-way ANOVA followed by the Newman–Keuls multiple comparison test (** *p* < 0.01, *** *p* < 0.001, versus vehicle; °°° *p* < 0.001 versus 10 μM PEA; ^§^ *p* < 0.05, ^§§§^ *p* < 0.001, versus 20 μM PEA).

To evaluate whether the drug-carrier conjugate GLUPEA, once inside the cells, is hydrolyzed and subsequently able to release PEA, we used the analysis system for the quantitative determination of aliamides. We measured the levels of PEA in the cell pellets of HaCaT keratinocytes treated for 40 min, 6 h, and 24 h with GLUPEA (10 or 20 μM), and compared them with those measured after treatment with chemically unmodified PEA at the same concentrations. We observed that both tested concentration of GLUPEA, in a concentration-dependent manner, were able to release PEA in HaCaT keratinocytes treated for 40 min and 6 h compared to vehicle-treated HaCaT keratinocytes (Figure 1), whereas only 20 μM GLUPEA was able to release PEA in HaCaT keratinocytes treated for 24 h (Figure 1). We also observed that, when HaCaT keratinocytes were treated with PEA (10 or 20 μM), its levels only increased in a concentration-dependent manner after 40 min of treatment compared to vehicle-treated HaCaT keratinocytes (Figure 1). The PEA levels also increased after 6 h of treatment with 20 μM PEA (Figure 1), whereas no significant increase was observed after 24 h of treatment with PEA at both concentrations (10 or 20 μM) (Figure 1). In addition, we observed that, when HaCaT keratinocytes were treated for 40 min and 24 h with GLUPEA (10 μM), the PEA levels were no higher than those detected in HaCaT keratinocytes after treatment with the same concentration of PEA (10 μM) (Figure 1). On the other hand, when HaCaT keratinocytes were treated for 40 min and 24 h with 20 μM GLUPEA, the PEA levels were 2-fold higher than those detected in HaCaT keratinocytes after treatment with the same concentration of PEA (20 μM) (Figure 1). Furthermore, when HaCaT keratinocytes were treated for 6 h with 10 μM GLUPEA, PEA levels were 12-fold higher than those detected in HaCaT keratinocytes after treatment with the same concentration of PEA (10 μM) (Figure 1). Finally and most importantly, when HaCaT keratinocytes were treated for 6 h with 20 μM GLUPEA, PEA levels were enhanced up to 27-fold compared to PEA levels measured in HaCaT keratinocytes after treatment with the same concentration of PEA (20 μM) (Figure 1). 

### 3.2. Glucuronic Acid Does Not Increase Endogenous PEA Levels in HaCaT Keratinocytes

**Figure 2:**
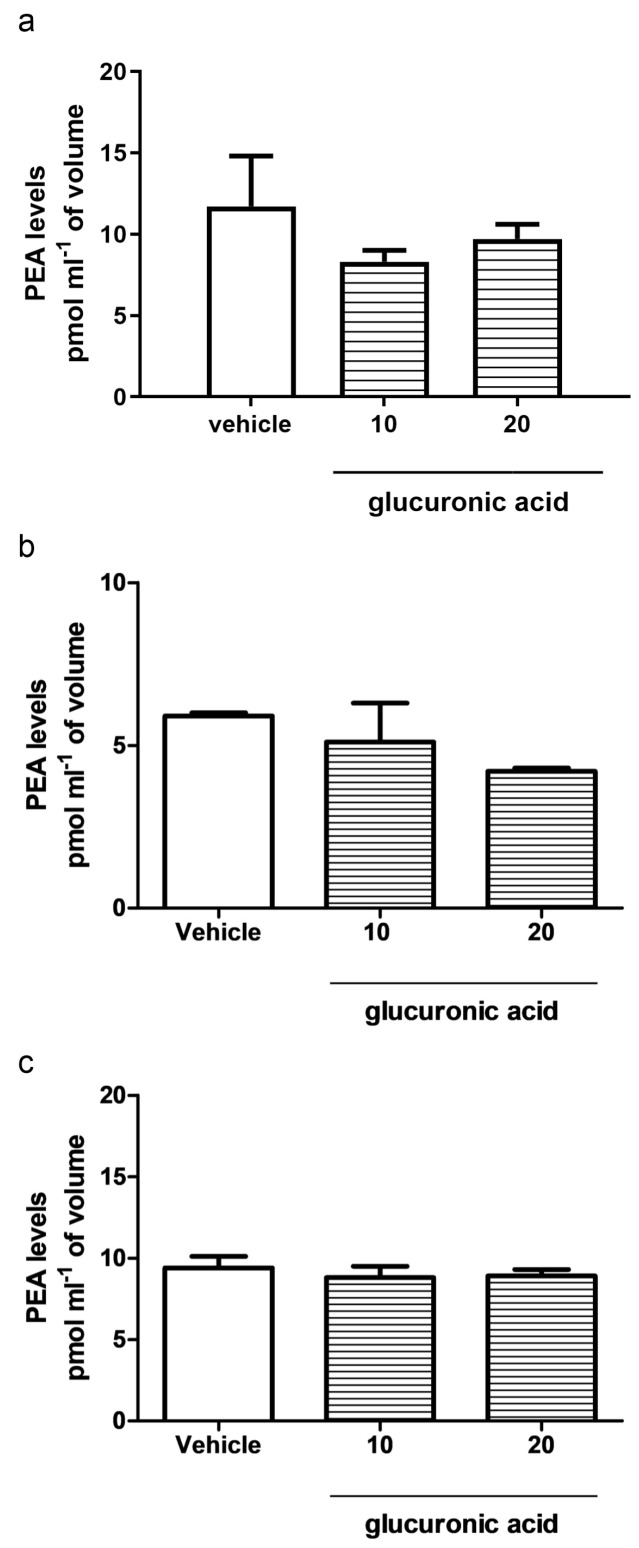
PEA levels in HaCaT keratinocytes after glucuronic acid treatment. PEA levels were measured in HaCaT keratinocytes treated for 40 min (**a**), 6 h (**b**), and 24 h (**c**) with glucuronic acid (10–20 μM). Data are representative of *n* = 4 separate determinations (means + s.e.m.). Statistical analyses were carried out using one-way ANOVA followed by the Newman–Keuls multiple comparison test.

To investigate whether glucuronic acid was able to induce an “entourage” effect on the endogenous levels of PEA in HaCaT keratinocytes, we measured the levels of PEA in the cells and supernatants after 40 min, 6 h, and 24 h of glucuronic acid treatment (10 or 20 μM). We observed that either concentrations of glucuronic acid were unable to increase the endogenous levels of PEA in HaCaT keratinocytes treated for 40 min, 6 h, and 24 h compared to vehicle-treated HaCaT keratinocytes (Figure 2). These results indicate that glucuronic acid produced from the hydrolysis of GLUPEA does not contribute to increasing the levels of PEA in HaCaT keratinocytes after GLUPEA treatment.

### 3.3. GLUPEA Increases Endogenous 2-AG Levels in HaCaT Keratinocytes 

**Figure 3:**
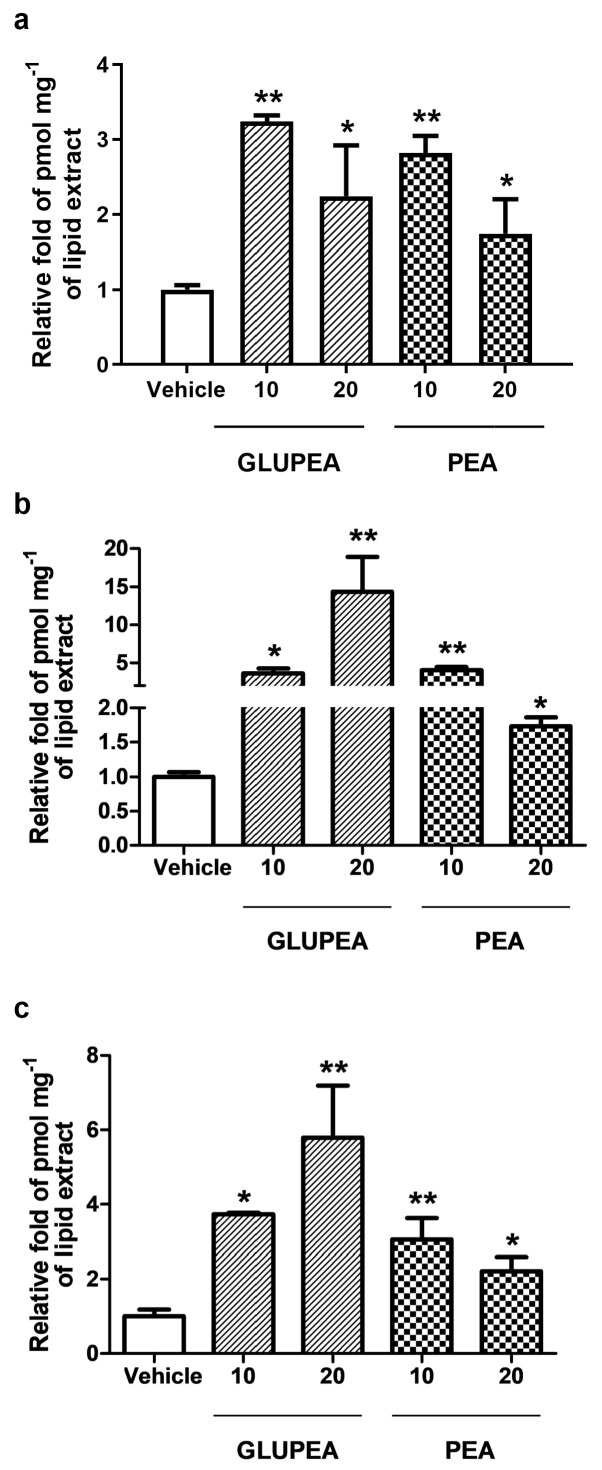
2-AG levels in HaCaT keratinocytes after GLUPEA treatment. 2-AG levels were measured in HaCaT keratinocytes treated for 40 min (**a**), 6 h (**b**), and 24 h (**c**) with GLUPEA (10–20 μM) or PEA (10–20 μM). Data are representative of *n* = 8 separate determinations (means + s.e.m.) and expressed as the relative fold of pmol mg^−1^ of lipid extract after normalizing for vehicle-treated cell values, considered as = 1. 2-AG levels measured in vehicle-treated cells were 8.32 ± 0.53 (**a**), 0.82 ± 0.06 (**b**), and 0.55 ± 0.10 (**c**). Statistical analyses were carried out using one-way ANOVA followed by the Newman–Keuls multiple comparison test (* *p* < 0.05, ** *p* < 0.01, versus vehicle).

To investigate whether GLUPEA was able to reproduce a typical effect of PEA, i.e., an “entourage” effect on the endogenous levels of 2-AG, we measured the levels of this endocannabinoid in the cells and supernatants of HaCaT keratinocytes after 40 min, 6 h, and 24 h of GLUPEA treatment (10 or 20 μM), and compared them with those measured after treatment with chemically unmodified PEA at the same concentrations (10 or 20 μM). In agreement with our previous data, the 2-AG levels were increased after 40 min, 6 h, and 24 h of treatment with PEA at both concentrations (10 or 20 μM) compared to vehicle-treated HaCaT keratinocytes (Figure 3). In addition, we observed that the 2-AG levels were increased after 40 min, 6 h, and 24 h of treatment with GLUPEA at both concentrations (10 or 20 μM) compared to vehicle-treated HaCaT keratinocytes (Figure 3). We also observed that after 40 min, 6 h, and 24 h of treatment with 10 μM GLUPEA, as well as after 40 min and 24 h of treatment with 20 μM GLUPEA, the levels of 2-AG were only slightly higher than basal levels (in a range from ~ 2.5- to 5.0-fold) (Figure 3). On the contrary, after 6 h of treatment with 20 μM GLUPEA, the levels of 2-AG were significantly higher than basal levels (by ~14-fold) (Figure 3b). Finally, and most importantly, after 40 min, 6 h, and 24 h of treatment with 10 μM GLUPEA, as well as after 40 min of treatment with 20 μM GLUPEA, the levels of 2-AG were no higher than those measured in HaCaT keratinocytes after treatment with the same concentrations of PEA (10 or 20 μM) (Figure 3). Conversely, after 6 and 24 h of treatment with 20 μM GLUPEA, the levels of 2-AG were 8-fold and 3-fold higher, respectively, than those measured in HaCaT keratinocytes after treatment with the same concentration of PEA (20 μM) (Figure 3b,c). 

### 3.4. GLUPEA Enhances 2-AG-Induced TRPV1 Channel Activation and Desensitization in HEK-293 Transfected with TRPV1 Channels

**Figure 4:**
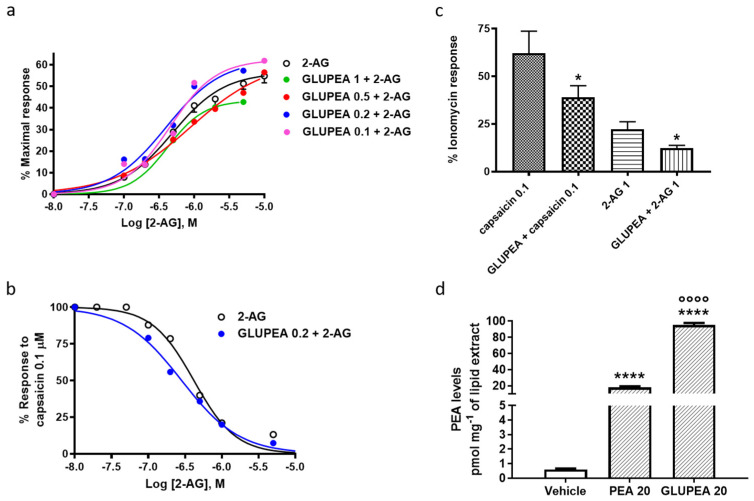
Effect of GLUPEA on 2-AG-induced TRPV1 channel activation and desensitization in human recombinant (HEK)-TRPV1 cells (**a**) Effect of GLUPEA (0.1, 0.2, 0.5, 1 μM) on the intracellular Ca^2+^ elevation by 2-AG in HEK-TRPV1 cells. GLUPEA was pre-incubated with cells 5 min prior to 2-AG. (**b**) Effect of GLUPEA (0.2 μM) on 2-AG-induced TRPV1 channel desensitization of the effect of capsaicin (0.1 μM) in HEK-TRPV1 cells. GLUPEA was pre-incubated with cells 5 min prior to 2-AG treatment. The effect on the intracellular Ca^2+^ of capsaicin (0.1 μM) alone was set as 100%. Data are representative of *n* = 4 separate determinations (means ± s.e.m.). (**c**) Effect of GLUPEA (20 μM) on the intracellular Ca^2+^ elevation by capsaicin (0.1 μM) or 2-AG (1 μM) in HEK-TRPV1 cells. HEK-TRPV1 cells were untreated or pre-treated for 6 h with GLUPEA (20 μM). Data are representative of *n* = 4 separate determinations (means ± s.e.m.). Statistical analyses were carried out using the Student’s *t* test. (* *p* < 0.05, versus capsaicin 0.1 or versus 2-AG 1 μM). (**d**) PEA levels were measured in HEK-TRPV1 cells treated for 6 h with GLUPEA (20 μM) or PEA (20 μM). Data are representative of *n* = 8 separate determinations (means + s.e.m.). Statistical analyses were carried out using one-way ANOVA followed by the Newman–Keuls multiple comparison test (**** *p* < 0.001, versus vehicle; °°°° *p* < 0.001, versus PEA 20 μM).

To investigate whether GLUPEA was able to reproduce another typical effect of PEA, i.e., the enhancement of 2-AG activation and desensitization of TRPV1-mediated intracellular Ca^2+^ elevation in HEK cells transfected with the human recombinant TRPV1 (HEK-TRPV1 cells), we measured the intracellular Ca^2+^ in these cells. In agreement with our previous data, 2-AG alone elevated intracellular Ca^2+^ in these cells (Figure 4a and Table 1), and also desensitized TRPV1 channels to the effect of capsaicin (0.1 μM) on intracellular Ca^2+^ (Figure 4b and Table 1). In addition, we observed that GLUPEA (0.1–0.2–0.5–1 μM) alone had no effect on the activation or desensitization of TRPV1 channels (data not shown). Conversely, GLUPEA only slightly increased the 2-AG activation of the TRPV1-mediated intracellular Ca^2+^ increase, with this effect only being statistically significant at 0.2 μM (Figure 4a and Table 1). On the other hand, GLUPEA strongly and significantly enhanced (by nearly 2-fold) the 2-AG-induced TRPV1 channel desensitization to the effect of 0.1 μM capsaicin (Figure 4b and Table 1). Finally, when HEK-TRPV1 cells were pre-treated for 6 h with GLUPEA (20 μM), the compound again significantly desensitized TRPV1 channels to the effect of both capsaicin (0.1 μM) and 2-AG (1 μM) (Figure 4c).

### 3.5. GLUPEA Releases PEA in HEK-TRPV1 Cells

We also measured the levels of PEA in HEK-TRPV1 cells treated for 6 h with GLUPEA (20 μM), and compared them with those measured after treatment with chemically unmodified PEA at the same concentration (20 μM). We observed that GLUPEA was able to release PEA in HEK-TRPV1 cells treated for 6 h compared to vehicle-treated HEK-TRPV1 cells (Figure 4d). Moreover, we observed that the PEA levels after treatment with GLUPEA (20 μM) were 5-fold higher than those measured in HEK-TRPV1 cells after treatment with the same concentration of PEA (20 μM) (Figure 4d).

### 3.6. GLUPEA Reduces MCP-2 Chemokine Levels in Poly-(I:C)-Stimulated HaCaT Keratinocytes

**Figure 5:**
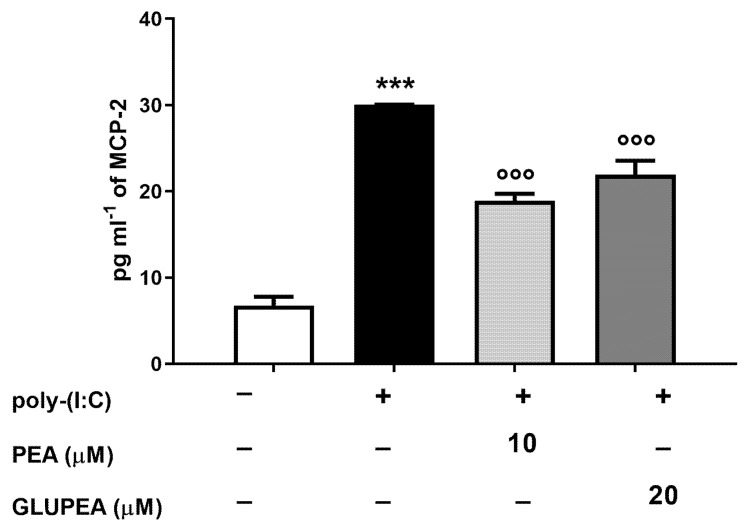
Effect of GLUPEA on MCP-2 chemokine levels in polyinosinic-polycytidylic acid (poly-(I:C))-induced allergic contact dermatitis (ACD) in HaCaT keratinocytes. MCP-2 chemokine levels were measured in the supernatants of poly-(I:C)-stimulated HaCaT cells (100 μg·mL^−1^, 6 h, 37 °C) in the presence of vehicle, PEA (10 μM), or GLUPEA (20 μM). Data are representative of *n* = 6 separate determinations (means + s.e.m.). Statistical analyses were carried out using one-way ANOVA followed by the Newman–Keuls multiple comparison test (*** *p* < 0.001 versus vehicle (i.e., cells without ACD); °°° *p* < 0.001 versus poly-(I:C)).

To investigate the effect of GLUPEA on poly-(I:C)-induced ACD in HaCaT keratinocytes, we measured the MCP-2 chemokine levels in keratinocytes stimulated for 6 h with poly-(I:C) (100 μg·mL^−1^) and treated with GLUPEA (20 μM), and compared them with those measured after treatment with the efficacious concentration of PEA (not chemically modified)—10 μM. In agreement with our previous data, HaCaT keratinocytes stimulated for 6 h with poly-(I:C) and treated with the vehicle of compounds produced significantly higher levels of MCP-2 chemokine compared to vehicle-stimulated HaCaT cells (Figure 5). Moreover, when HaCaT keratinocytes were co-stimulated with poly-(I:C) and 10 μM PEA, the levels of MCP-2 chemokine were strongly reduced compared to poly-(I:C)-stimulated HaCaT keratinocytes treated with the vehicle of PEA (Figure 5). Likewise, we observed that when HaCaT keratinocytes were co-stimulated with poly-(I:C) and GLUPEA, at the highest concentration tested (20 μM), the levels of MCP-2 chemokine were also reduced compared to poly-(I:C)-stimulated HaCaT keratinocytes treated with the vehicle of GLUPEA (Figure 5). The effect of 20 μM GLUPEA in poly-(I:C)-stimulated HaCaT keratinocytes was comparable to that observed with 10 μM PEA (Figure 5). No significant variation was observed in MCP-2 chemokine levels after HaCaT cells were treated with GLUPEA alone (at the highest concentration tested, 20 µM), i.e., in the absence of poly-(I:C), compared to vehicle-treated HaCaT cells (data not shown).

### 3.7. GLUPEA Reduces the Signs of Colitis in DNBS-Injected Mice

**Figure 6:**
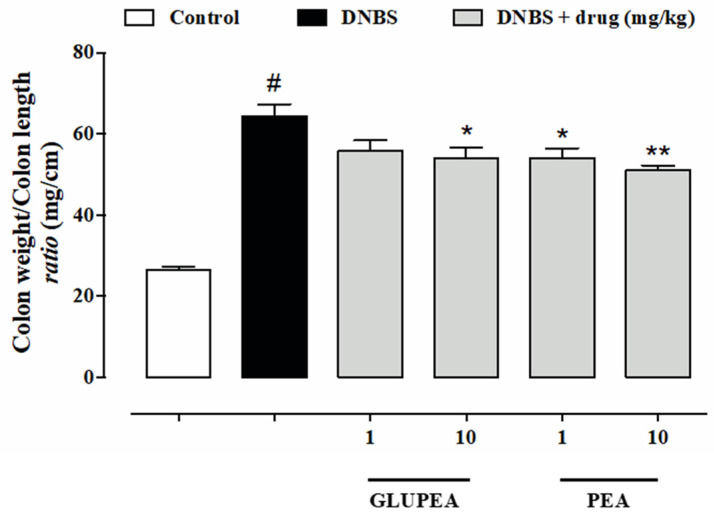
Effect of GLUPEA on the colon weight/colon length ratio in dinitrobenzene sulfonic acid (DNBS)-induced colitis in mice. Changes in the colon weight/colon length ratio from control and DNBS-injected mice in the presence or absence of orally administrated PEA-um (1–10 mg·kg^−1^) or GLUPEA (1–10 mg·kg^−1^). Mice were weighed before DNBS (or vehicle) injection and immediately before killing. Tissues were analysed 3 days after vehicle or DNBS injection. PEA-um (1–10 mg kg^−1^) or GLUPEA (1–10 mg kg) was administered by oral gavage once a day for three consecutive days starting 24 h after the inflammatory insult. Data are the mean + s.e.m. of a single experiment, with 15 mice for each experimental group. Statistical analyses were carried out using one-way ANOVA followed by Dunnett’s multiple comparisons test (^#^ *p* < 0.0001 versus control (i.e., mice without intestinal inflammation); * *p* < 0.05, ** *p* < 0.01, versus DNBS).

To investigate the effect of GLUPEA in vivo, we selected a mouse model of colitis, i.e., DNBS-induced colon inflammation, in which PEA was previously found to be protective [57]. We evaluated colonic inflammation, measured by the colon weight/colon length ratio (a simple and reliable marker of intestinal inflammation and/or damage), in mice after an intracolonic injection of DNBS and the administration of GLUPEA (1–10 mg kg^−1^). The effect of GLUPEA was compared to that evaluated after the administration of PEA-um (not chemically modified) at the same doses of 1–10 mg kg^−1^. In agreement with our previous data, the intracolonic DNBS injection produced inflammatory damage, as shown by the approximately 2.5-fold increase in the colon weight/colon length ratio (Figure 6). In addition, we observed that PEA-um (1–10 mg kg^−1^), orally administered following the inflammatory insult, in a dose-dependent manner, decreased the effect of DNBS on the colon weight/colon length ratio (Figure 6). Likewise, orally administered GLUPEA (at the same doses of 1–10 mg kg^−1^) was as efficacious as PEA-um at reducing the effect of DNBS on the colon weight/colon length ratio (Figure 6).

### 3.8. GLUPEA Reduces MPO Activity

**Figure 7:**
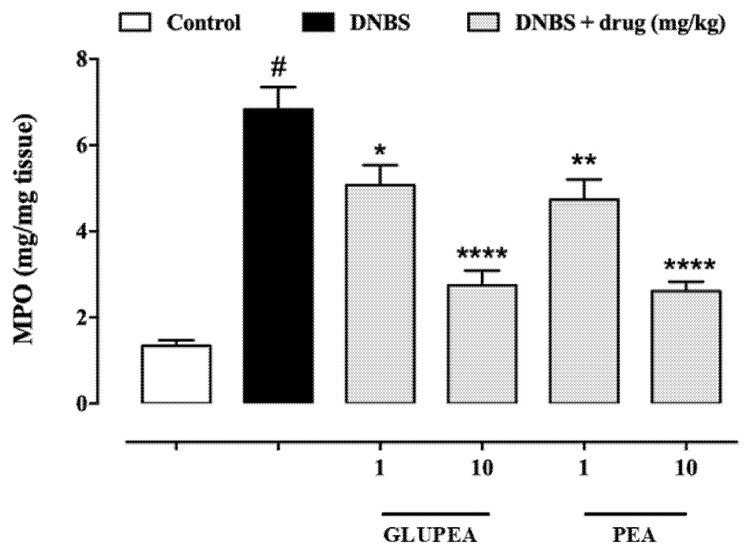
Effect of GLUPEA on the MPO activity in DNBS-induced colitis in mice. MPO activity was measured on colonic tissues 3 days after vehicle or DNBS injection. PEA-um (1–10 mg kg^−1^), or GLUPEA (1–10 mg kg^−1^) was administered by oral gavage once a day for three consecutive days starting 24 h after the inflammatory insult. Data are the mean + s.e.m. of a single experiment, with 15 mice for each experimental group. Statistical analyses were carried out using one-way ANOVA followed by Dunnett’s multiple comparisons test (^#^ *p* < 0.0001 versus control (i.e., mice without intestinal inflammation); * *p* < 0.05, ** *p* < 0.01, **** *p* < 0.001, versus DNBS).

To confirm the anti-inflammatory activity of GLUPEA in vivo, we measured the MPO activity (a marker of neutrophil infiltration in the intestinal inflammation) in mice after an intracolonic injection of DNBS and the administration of GLUPEA (1–10 mg kg^−1^). Additionally, in this case, the effect of GLUPEA was compared to that evaluated after the administration of PEA-um (not chemically modified) at the same doses of 1–10 mg kg^−1^. In agreement with our previous data, DNBS-induced colitis was associated with a significant increase in MPO activity (Figure 7). In addition, we observed that orally administered PEA-um (1–10 mg kg^−1^), strongly and in a dose-dependent manner, reduced MPO activity (Figure 7). Orally administered GLUPEA (at the same doses of 1–10 mg kg^−1^) was as efficacious as PEA-um at reducing the MPO activity (Figure 7). 

## 4. Discussion

In this study, we synthesized and tested GLUPEA, which is a drug-carrier conjugate between the nutraceutical and endogenous lipid mediator, PEA, and glucuronic acid as the carrier, in an attempt to develop the first pro-drug of PEA. The ability of GLUPEA to deliver PEA and induce an “entourage” effect on the endogenous levels of 2-AG, as well as indirectly activate TRPV1 channels, was demonstrated in in vitro cell systems. In addition, the potential anti-inflammatory activity of GLUPEA was also demonstrated in experimental models of ACD in HaCaT keratinocytes and colitis in mice.

First, we evaluated PEA release from the drug-carrier conjugate GLUPEA by measuring the levels of PEA in the cell pellets of HaCaT keratinocytes stimulated with GLUPEA. We found that the administration of GLUPEA released PEA in a concentration-dependent manner and up to 6 h after cell treatment. Only the highest concentration of the compound also released PEA to the cells 24 h after administration. Importantly, we found that the administration of GLUPEA resulted in a better delivery of PEA compared to unmodified PEA administered at the same concentration. This considerable difference might be due to the fact that PEA is easily and rapidly degraded into palmitic acid and ethanolamine by the action of two hydrolytic enzymes—FAAH and NAAA [21,22]—which are expressed in HaCaT keratinocytes [15,37]. On the other hand, PEA conjugation with glucuronic acid might, at the same time, protect PEA from degradation by FAAH and NAAA, and release PEA to cells following hydrolysis by esterases, thereby resulting in an increase of the exogenous PEA delivery to the cell. Importantly, glucuronic acid *per se* did not enhance the endogenous levels of PEA in HaCaT keratinocytes, indicating that GLUPEA only elevates the levels of PEA via the release of this compound and not because of any effect of the other product of its hydrolysis, i.e., glucuronic acid. 

We recently proved that PEA is able to increase the endogenous levels of 2-AG in HaCaT keratinocytes (by ~3-fold), as well as in human and canine plasma (by ~2-fold and ~20-fold, respectively) [37]. Therefore, we investigated whether GLUPEA was able to produce the same effect in HaCaT keratinocytes. We found that the endogenous levels of 2-AG increased after GLUPEA treatment, and this increase was the strongest (~14-fold higher than basal levels) after 6 h of the administration of 20 μM GLUPEA, i.e., when the increase in PEA concentrations (~70-fold higher than basal levels) caused by the same treatment was also the strongest. Importantly, we found that after 6 h of the administration of 20 μM GLUPEA, the increase in endogenous 2-AG levels was also stronger (~8-fold) compared to that found after unmodified PEA administration at the same concentration. These findings suggest that PEA, delivered to cells following GLUPEA administration and hydrolysis, is able to induce a concentration-dependent “entourage” effect on the endogenous levels of 2-AG in HaCaT keratinocytes.

Next, we investigated the mechanism of action and molecular targets of GLUPEA. In particular, we focused on TRPV1 channels, since we recently demonstrated that PEA (at a concentration of 2 μM) enhances 2-AG-induced TRPV1 channel activation and desensitization, in HEK-TRPV1 cells [37]. Therefore, we studied the effect of GLUPEA on TRPV1 channels in the presence or absence of 2-AG, in HEK-TRPV1 cells. We observed that GLUPEA *per se*, as previously found for PEA, had no effect on the activation or desensitization of TRPV1 channels. Conversely, GLUPEA (at the concentration of 0.2 μM) slightly increased the 2-AG activation of TRPV1 channels, but significantly enhanced 2-AG-induced TRPV1 channel desensitization. Importantly, the effect of 0.2 μM GLUPEA on 2-AG-induced TRPV1 channel activation and desensitization was comparable to that observed with PEA administered at a concentration 10-fold higher (2 μM) [37]. Increased PEA levels (5-fold higher) were observed in HEK-TRPV1 cells treated with GLUPEA compared to those observed in HEK-TRPV1 cells treated with PEA at the same concentration, thus indicating that these cells are also able convert the pro-drug into PEA. In order to demonstrate this latter effect, however, we had to use a higher dose (20 μM) of, and a longer incubation period (6 h) with, GLUPEA than those used to observe the enhanced 2-AG-mediated desensitization, although these conditions were identical to those necessary to observe the maximal pro-drug effect in HaCaT cells, and required due to the sensitivity limit of our PEA detection method. Therefore, we can only assume, judging from the biological action observed, that a 100-fold smaller concentration of, and a ~300-fold shorter incubation with, GLUPEA of HEK-TRPV1 cells are also able to cause the release of sufficient local amounts of PEA to exert an “entourage” effect on the 2-AG desensitization of TRPV1. On the other hand, when we used 2-AG (or capsaicin)-induced TRPV1 desensitization assay conditions similar to those necessary (because of the detection limit of our LC-MS assay) to measure PEA release from GLUPEA in HEK-TRPV1 cells, i.e., a longer pre-incubation and a higher concentration of the compound, we could still observe that GLUPEA enhanced agonist-induced TRPV1 desensitization. Therefore, altogether, our data obtained in in vitro cell systems suggest that GLUPEA, once inside cells, delivers PEA, which is able to increase the endogenous levels of endocannabinoid 2-AG (plausibly through the stimulation of DAGL, [29]), thereby inducing TRPV1 channel desensitization. Importantly, compared to PEA, a lower concentration of GLUPEA was able to potentiate 2-AG-induced TRPV1 channel desensitization in vitro.

We have recently demonstrated that PEA attenuates allergic inflammation in an in vitro model of the first phase of ACD in HaCaT keratinocytes [15], and that PEA-um improves inflammation and intestinal permeability in an in vivo model of colitis in mice [57]. In particular, we proved that PEA was capable of decreasing the expression and release of the pro-inflammatory chemokine MCP-2 (mainly produced by activated keratinocytes) in HaCaT keratinocytes when stimulated with an agonist of the toll-like receptor 3—poly-(I:C) [15]. Likewise, orally administered PEA-um was able to reduce the colon weight/colon length ratio (a marker of intestinal inflammation and/or damage) of inflamed colonic tissue, as well as the MPO activity (a marker of neutrophil infiltration largely used to measure intestinal inflammation), in DNBS-induced colitis in mice [57]. Based on these data, we considered whether GLUPEA was also able to inhibit the allergic inflammatory reaction in keratinocytes and colitis in mice. Indeed, we found that GLUPEA reduces the concentrations of MCP-2 in stimulated HaCaT keratinocytes and that this effect was mainly similar to that observed in response to PEA. Finally, we demonstrated that a single dose of orally administered GLUPEA was as efficacious as the same dose of PEA-um at reducing the colon weight/colon length ratio and MPO activity in DNBS-induced colitis in mice.

## 5. Conclusions

We have demonstrated that (1) glucuronic acid is a good starting point for developing new drug-carrier conjugates with highly lipophilic compounds, such as PEA; (2) the drug-carrier conjugate developed here between PEA and glucuronic acid, called GLUPEA, efficiently delivers PEA to cells; (3) like PEA, and possibly by acting as a source of it, GLUPEA increases the endogenous levels of endocannabinoid 2-AG and increases 2-AG-induced TRPV1 channel desensitization; and (4) like PEA, and possibly by acting as a source of it, GLUPEA inhibits the production of the chemokine MCP-2 in an in vitro model of ACD, and inflammation in an in vivo model of colitis. Therefore, GLUPEA represents a promising drug delivery system for PEA, and a potential anti-inflammatory bioregulator. 

## Figures and Tables

**Table 1:** Effects of GLUPEA on elevation of intracellular Ca^2+^ through TRPV1 channels induced by 2-AG.

	EC_50_	IC_50_ (vs. Capsaicin 0.1 μM)	% Efficacy (vs. Ionomycin 4 μM)
2-AG	0.48 ± 0.04	0.42 ± 0.02	56.4 ± 1.0
GLUPEA 1.0 μM + 2-AG	0.42 ± 0.01	1.07 ± 0.06	43.4 ± 0.1
GLUPEA 0.5 μM + 2-AG	0.89 ± 0.14	0.43 ± 0.03	62.2 ± 3.1
GLUPEA 0.2 μM + 2-AG	0.40 ± 0.08	0.28 ± 0.01 **	62.3 ± 5.0
GLUPEA 0.1 μM + 2-AG	0.45 ± 0.05	0.38 ± 0.01	62.4 ± 1.4

The EC_50_ values (μM) shown are for 2-AG alone and after GLUPEA pre-incubation for 5 min before 2-AG. The IC_50_ values (mM) shown are for TRPV1 channel desensitization by 2-AG alone and in the presence of GLUPEA, pre-incubated 5 min before capsaicin (0.1 μM). The efficacy of the compounds or their combinations is reported as % of the maximum effect observed with ionomycin (4 mM). All the experiments were performed in HEK-TRPV1 cells. Data are representative of *n* = 4 separate determinations (means ± s.e.m.). Statistical analyses were carried out using one-way ANOVA followed by Bonferroni’s multiple comparison test (** *p* < 0.01, versus 2-AG alone).

## Data Availability

The authors declare that the data supporting the findings of this study are available within the article.

## Abbreviations

2-AG: 2-arachidonoyl-glycerol; ACD, allergic contact dermatitis; DNBS, dinitrobenzene sulfonic acid; HEK-293, human embryonic kidney; HaCaT, human keratinocyte; MCP-2, monocyte chemoattractant protein 2; MPO, myeloperoxidase; Poly-(I:C), polyinosinic-polycytidylic acid; TRPV1, transient receptor potential vanilloid type 1.
